# Supplementary material for: HIVprotI: an integrated web based platform for prediction and design of HIV proteins inhibitors
Source: J Cheminform. 2018 Mar 9;10:12. doi: 10.1186/s13321-018-0266-y (PMC5845081; doi:10.1186/s13321-018-0266-y)
Supplement: Supplementary file 2 — Additional file 2. Source code of HIVProtI web server. [file 13321_2018_266_MOESM2_ESM.zip › HIVprotI_Source-code/hivprotI/weka-3-6-6/doc/weka/core/SingleIndex.html]

SingleIndex


---


|  |  |  |
| --- | --- | --- |
| **Overview** \| **Package** \| **Class** \| **Tree** \| **Deprecated** \| **Index** \| **Help** \| **Weka's home** | |  |
| **PREV CLASS**   **NEXT CLASS** | **FRAMES**    **NO FRAMES**     **All Classes** |  |
| SUMMARY: NESTED \| FIELD \| CONSTR \| METHOD | DETAIL: FIELD \| CONSTR \| METHOD |  |


---


## weka.core Class SingleIndex

```
java.lang.Object
  weka.core.SingleIndex
```

**All Implemented Interfaces:**: java.io.Serializable, RevisionHandler

---

``` public class SingleIndex extends java.lang.Object implements java.io.Serializable, RevisionHandler ```

Class representing a single cardinal number. The number is set by a
string representation such as:

`first
last
1
3` 

The number is internally converted from 1-based to 0-based (so methods that
set or get numbers not in string format should use 0-based numbers).

**Version:**
:   $Revision: 1.8 $

**Author:**
:   Eibe Frank (eibe@cs.waikato.ac.nz)

**See Also:**: Serialized Form

---

**Constructor Summary**

`SingleIndex()`
  
          Default constructor.

`SingleIndex(java.lang.String index)`
  
          Constructor to set initial index.


| **Method Summary** | |
| --- | --- |
| `int` | `getIndex()`             Gets the selected index |
| `java.lang.String` | `getRevision()`             Returns the revision string. |
| `java.lang.String` | `getSingleIndex()`             Gets the string representing the selected range of values |
| `static java.lang.String` | `indexToString(int index)`             Creates a string representation of the given index. |
| `static void` | `main(java.lang.String[] argv)`             Main method for testing this class. |
| `void` | `setSingleIndex(java.lang.String index)`             Sets the index from a string representation. |
| `void` | `setUpper(int newUpper)`             Sets the value of "last". |
| `java.lang.String` | `toString()`             Constructs a representation of the current range. |

**Methods inherited from class java.lang.Object**

`equals, getClass, hashCode, notify, notifyAll, wait, wait, wait`

**Constructor Detail**

### SingleIndex

```
public SingleIndex()
```

:   Default constructor.

---


### SingleIndex

```
public SingleIndex(java.lang.String index)
```

:   Constructor to set initial index.

    **Parameters:**: `index` - the initial index **Throws:**: `java.lang.IllegalArgumentException` - if the index is invalid


**Method Detail**

### setUpper

```
public void setUpper(int newUpper)
```

:   Sets the value of "last".

    :   **Parameters:**: `newUpper` - the value of "last"

---


### getSingleIndex

```
public java.lang.String getSingleIndex()
```

:   Gets the string representing the selected range of values

    :   **Returns:**: the range selection string

---


### setSingleIndex

```
public void setSingleIndex(java.lang.String index)
```

:   Sets the index from a string representation. Note that setUpper()
    must be called for the value to be actually set

    :   **Parameters:**: `index` - the index set **Throws:**: `java.lang.IllegalArgumentException` - if the index was not well formed

---


### toString

```
public java.lang.String toString()
```

:   Constructs a representation of the current range. Being a string
    representation, the numbers are based from 1.

    :   **Overrides:**: `toString` in class `java.lang.Object`
    :   **Returns:**: the string representation of the current range

---


### getIndex

```
public int getIndex()
```

:   Gets the selected index

    :   **Returns:**: the selected index **Throws:**: `java.lang.RuntimeException` - if the upper limit of the index hasn't been defined

---


### indexToString

```
public static java.lang.String indexToString(int index)
```

:   Creates a string representation of the given index.

    :   **Parameters:**: `index` - the index to turn into a string. Since the index will typically come from a program, indices are assumed from 0, and thus will have 1 added in the String representation. **Returns:**: the string representation

---


### getRevision

```
public java.lang.String getRevision()
```

:   Returns the revision string.

    :   **Specified by:**: `getRevision` in interface `RevisionHandler`
    :   **Returns:**: the revision

---


### main

```
public static void main(java.lang.String[] argv)
```

:   Main method for testing this class.

    :   **Parameters:**: `argv` - one parameter: a test index specification


---


|  |  |  |
| --- | --- | --- |
| **Overview** \| **Package** \| **Class** \| **Tree** \| **Deprecated** \| **Index** \| **Help** \| **Weka's home** | |  |
| **PREV CLASS**   **NEXT CLASS** | **FRAMES**    **NO FRAMES**     **All Classes** |  |
| SUMMARY: NESTED \| FIELD \| CONSTR \| METHOD | DETAIL: FIELD \| CONSTR \| METHOD |  |


---
